# Improving the management of open tibia fractures, Malawi

**DOI:** 10.2471/BLT.23.290755

**Published:** 2024-02-29

**Authors:** Alexander Thomas Schade, Maureen Sabawo, Zahra Jaffry, Nohakhelha Nyamulani, Chikumbutso Clara Mpanga, Leonard Banza Ngoie, Andrew John Metcalfe, David Graham Lalloo, William James Harrison, Andrew Leather, Peter MacPherson

**Affiliations:** a Public Health Department, Malawi-Liverpool-Wellcome Trust, Queen Elizabeth Central Hospital, P.O. Box 30096, Blantyre, Malawi.; b Department of Health Systems and Policy, Kamuzu University of Health Sciences, Blantyre, Malawi.; c Trauma and Orthopaedic Department, Bart’s Health NHS Trust, London, England.; d Trauma and Orthopaedic Department, Queen Elizabeth Central Hospital, Blantyre, Malawi.; e Trauma and Orthopaedic Department, Kamuzu Central Hospital, Lilongwe, Malawi.; f Clinical Trials Unit, University of Warwick Medical School, Coventry, England.; g Liverpool School of Tropical Medicine, Liverpool, England.; h AO Alliance, Davos, Switzerland.; i King's Global Health Partnerships, King’s College London, London, England.; j School of Health and Wellbeing, University of Glasgow, Glasgow, Scotland.

## Abstract

**Objective:**

To assess the impact of an open fracture intervention bundle on clinical management and patient outcomes of adults in Malawi with open tibia fractures.

**Methods:**

We conducted a before-and-after implementation study in Malawi in 2021 and 2022 to assess the impact of an open fracture intervention bundle, including a national education course for clinical officers and management guidelines for open fractures. We recruited 287 patients with open tibia fractures. The primary outcome was a before-and-after comparison of the self-reported short musculoskeletal function assessment score, a measure of patient function. Secondary outcomes included clinical management; and clinician knowledge and implementation evaluation outcomes of 57 health-care providers attending the course. We also constructed multilevel regression models to investigate associations between clinical knowledge, patient function, and implementation evaluation before and after the intervention.

**Findings:**

The median patient function score at 1 year was 6.8 (interquartile range, IQR: 1.5 to 14.5) before intervention and 8.4 (IQR: 3.8 to 23.2) after intervention. Compared with baseline scores, we found clinicians’ open fracture knowledge scores improved 1 year after the intervention was implemented (mean posterior difference: 1.6, 95% highest density interval: 0.9 to 2.4). However, we found no difference in most aspects of clinicians’ open fracture management practice.

**Conclusion:**

Despite possible improvement in clinician knowledge and positive evaluation of the intervention implementation, our study showed that there was no overall improvement in clinical management, and weak evidence of worsening patient function 1 year after injury, after implementation of the open fracture intervention bundle.

## Résumé

**Objectif:**

Évaluer l'impact d'un ensemble d'interventions relatives aux fractures ouvertes sur la prise en charge clinique et la santé des patients adultes souffrant d'une fracture ouverte du tibia au Malawi.

**Méthodes:**

Afin d'évaluer cet impact, nous avons mené une étude dans le pays en 2021 et 2022, avant et après la mise en œuvre, qui prévoyait un cours d'éducation national destiné aux responsables cliniques et des directives de prise en charge des fractures ouvertes. Nous avons recruté 287 patients présentant des fractures ouvertes du tibia. Une comparaison avant/après de la courte note d'évaluation de la fonction musculosquelettique autodéclarée, qui permet de déterminer à quel point le patient est opérationnel, constituait le critère d'évaluation principal. Les critères d'évaluation secondaires incluaient la prise en charge clinique, ainsi que les résultats de l'évaluation des connaissances cliniques et de la mise en œuvre pour 57 prestataires de soins de santé ayant participé au cours. Nous avons également élaboré des modèles de régression multiniveaux en vue d'examiner les liens entre les connaissances cliniques, le degré opérationnel du patient et l'évaluation de la mise en œuvre avant et après l'intervention.

**Résultats:**

La note médiane de la fonction des patients à un an s'élevait à 6,8 (écart interquartile, EI: 1,5 à 14,5) avant l'intervention et à 8,4 (EI: 3,8 à 23,2) après l'intervention. En la comparant avec les notes initiales, nous avons découvert que les connaissances des médecins en matière de fractures ouvertes s'étaient améliorées un an après le déploiement de l'intervention (différence moyenne a posteriori: 1,6, intervalle de densité le plus élevé à 95%: 0,9 à 2,4). Toutefois, en ce qui concerne la prise en charge des fractures ouvertes par les médecins, nous n'avons observé aucune différence pour la plupart des aspects.

**Conclusion:**

Malgré une possible amélioration des connaissances médicales et une intervention dont la mise en œuvre a été jugée positive, notre étude a montré qu'il n'existait aucune amélioration globale de la prise en charge clinique et peu d'éléments indiquant une dégradation de l'état du patient un an après sa blessure une fois que l'ensemble des interventions relatives aux fractures ouvertes a été adopté.

## Resumen

**Objetivo:**

Evaluar el impacto de un paquete de intervención en fracturas abiertas sobre el manejo clínico y los resultados de los pacientes adultos en Malawi con fracturas abiertas de tibia.

**Métodos:**

Se realizó un estudio de implementación antes y después en Malawi entre 2021 y 2022 para evaluar el impacto de un paquete de intervención en fracturas abiertas, que incluye un curso nacional de educación para funcionarios clínicos y directrices de manejo para fracturas abiertas. Se reclutaron 287 pacientes con fracturas abiertas de tibia. El resultado primario fue una comparación antes y después del puntaje breve de evaluación de la función musculoesquelética autoinformada, una medida de la función del paciente. Los resultados secundarios incluyeron: el manejo clínico; y los resultados de la evaluación del conocimiento clínico y la implementación de 57 profesionales sanitarios que asistieron al curso. También se construyeron modelos de regresión multinivel para investigar las asociaciones entre el conocimiento clínico, la función del paciente y la evaluación de la implementación antes y después de la intervención.

**Resultados:**

El puntaje medio de la función del paciente a un año fue de 6,8 (rango intercuartílico, IQR: 1,5 a 14,5) antes de la intervención y de 8,4 (IQR: 3,8 a 23,2) después de la intervención. En comparación con los puntajes iniciales, se observó que los puntajes de conocimiento de fractura abierta de los médicos mejoraron un año después de la implementación de la intervención (diferencia posterior media: 1,6; intervalo de densidad más alta del 95%: 0,9 a 2,4). Sin embargo, no se observaron diferencias en la mayoría de los aspectos de la práctica clínica para el manejo de las fracturas abiertas.

**Conclusión:**

A pesar de la posible mejora de los conocimientos de los médicos y de la evaluación positiva de la implementación de la intervención, nuestro estudio mostró que no hubo una mejora general en el manejo clínico y pruebas débiles de empeoramiento de la función del paciente un año después de la lesión tras la implementación del paquete de intervención en fracturas abiertas.

## ملخص

الغرض تقييم تأثير حزمة التدخلات الخاصة بالكسور المفتوحة، على الإدارة الإكلينيكية ونتائج المرضى من البالغين المصابين بكسور مفتوحة في قصبة الساق في ملاوي.

الطريقة قمنا بإجراء دراسة لما قبل التنفيذ وبعده في ملاوي خلال الفترة من 2021 إلى 2022 لتقييم تأثير حزمة التدخلات الخاصة بالكسور المفتوحة، بما في ذلك تضمين دورة تعليمية وطنية للموظفين الإكلينيكيين، والمبادئ التوجيهية لإدارة الكسور المفتوحة. قمنا بالاستعانة بعدد 287 مريضًا يعانون من كسور مفتوحة في قصبة الساق. وكانت النتيجة الأولية مقارنة بين درجة تقييم وظيفة العضلات والعظام القصيرة المبلغ عنها ذاتيًا، قبل الدراسة وبعدها، وهو مقياس لحركة المريض. وشملت النتائج الثانوية: الإدارة الإكلينيكية؛ ومعرفة الأطباء الإكلينيكيين، ونتائج تقييم التنفيذ لعدد 57 من مقدمي الرعاية الصحية الذين حضروا الدورة. قمنا أيضًا ببناء نماذج تحوف متعددة المستويات للتحقيق في الارتباطات بين المعرفة الإكلينيكية، وحركة المريض، وتقييم التنفيذ قبل التدخل وبعده.

النتائج كان متوسط درجة حركة المريض في سنة واحدة هو 6.8 (المدى بين الشرائح الربيعية (IQR): 1.50 إلى 14.5) قبل التدخل، و8.4 (المدى بين الشرائح الربيعية: 3.8 إلى 23.2) بعد التدخل. بالمقارنة مع الدرجات الأساسية، وجدنا أن درجات معرفة الأطباء الإكلينيكيين بالكسور المفتوحة قد تحسنت بعد عام واحد من تنفيذ التدخل (متوسط الاختلاف اللاحق: 1.6، أعلى فاصل كثافة بنسبة %95: 0.9 إلى 2.4). ومع ذلك، لم نجد أي اختلاف في معظم جوانب ممارسات الأطباء الإكلينيكيين لإدارة الكسور المفتوحة.

الاستنتاج على الرغم من التحسن المحتمل في معرفة الطبيب الإكلينيكي، والتقييم الإيجابي لتنفيذ التدخل، أظهرت دراستنا أنه لم يكن هناك تحسن عام في الإدارة الإكلينيكية، وضعف الأدلة على تدهور حركة المريض بعد عام واحد من الإصابة، بعد تنفيذ حزمة التدخل في الكسر المفتوح.

## 摘要

**目的:**

评估开放性骨折干预措施对马拉维开放性胫骨骨折成人患者的临床诊治和患者结果的影响。

**方法:**

我们于 2021 年至 2022 年在马拉维进行了一项实施前后对比研究，以评估开放性骨折干预措施的影响，干预措施包括针对临床医生的国家教育课程和开放性骨折的诊治指南。我们招募了 287 名开放性胫骨骨折患者。主要结果是对患者自我报告的简明肌肉骨骼功能评估量表评分（一种衡量患者功能的指标）进行干预前后的比较。次要结果包括：临床诊治；参加课程的 57 名卫生保健服务提供者的临床知识和对其实施干预的评估结果。我们还构建了多层次回归模型，以研究实施干预前后临床知识、患者功能和实施干预的评估结果之间的关系。

**结果:**

实施干预 1 年前，患者功能评分的中位数为 6.8 分（四分位距，IQR：1.5 至 14.5），实施干预后为 8.4 分（IQR：3.8 至 23.2）。与基线评分相比，我们发现实施干预 1 年后临床医生的开放性骨折知识评分有所提高（后验均值差异：1.6，95% 最高密度区间：0.9 至 2.4）。然而，我们发现临床医生在开放性骨折诊治的大多数实践方面没有差异。

**结论:**

尽管临床医生的知识可能有所提高，对干预措施实施的评估也有较好的结果，但我们的研究显示，在实施开放式骨折干预措施 1 年后，临床诊治并没有得到全面改善，患者功能恶化的证据也很微弱。

## Резюме

**Цель:**

Оценить влияние комплекса мероприятий по лечению открытых переломов на клиническое ведение и результаты лечения взрослых в Малави с открытыми переломами большеберцовой кости.

**Методы:**

В период с 2021 по 2022 год в Малави было проведено исследование «до и после» с целью оценки влияния комплекса мероприятий по лечению открытых переломов, в том числе национального образовательного курса для ведущих специалистов и руководства по лечению открытых переломов. В исследовании приняли участие 287 пациентов с открытыми переломами большеберцовой кости. Главным результатом являлось сравнение показателей до и после краткой оценки функции опорно-двигательного аппарата со слов пациентов, то есть оценки функционального состояния пациента. К вторичным результатам относились: клиническое управление; оценка знаний и опыта 57 медицинских работников, прошедших курс. Также были построены многоуровневые регрессионные модели для изучения взаимосвязей между такими показателями, как знание течения и симптомов заболеваний, функциональное состояние пациента и оценка внедрения до и после вмешательства.

**Результаты:**

Медианный показатель оценки функционального состояния пациента через 1 год составил 6,8 (межквартильный интервал, МКИ: 1,5–14,5) до вмешательства и 8,4 (МКИ: 3,8–23,2) после вмешательства. По сравнению с показателями на исходном уровне было обнаружено, что через год после проведения вмешательства уровень знаний врачей об открытых переломах повысился (апостериорные сравнения средних: 1,6; 95%-й интервал наибольшей плотности: 0,9–2,4). Однако в большинстве аспектов практики лечения открытых переломов различий не обнаружено.

**Вывод:**

Несмотря на возможное повышение уровня знаний врачей и положительную оценку внедрения вмешательства, по результатам проведенного исследования было выявлено отсутствие общего улучшения клинического ведения и слабые доказательства ухудшения функционального состояния пациентов через 1 год после травмы в связи с внедрением комплекса вмешательств при открытом переломе.

## Introduction

Economic growth, urbanization and increased road vehicle use have led to a rapid rise in the rates of road traffic incidents in low-income countries.^1^ Open fractures are increasingly common following such incidents. These can cause substantial complications and disability, including infection, non-union of fractured bones, amputation and poor function.^2^ Standardized treatment includes antibiotics, debridement, early stabilization and wound coverage.^3^

In high-income countries, the implementation of set standards for managing open fractures has improved care quality and reduced the need for surgeries.^4^ Whether similar improvements can occur in low-income countries with the implementation of locally adapted guidelines remains unknown.^5^

Malawi, a low-income country in south-eastern Africa, has one of the highest rates of road traffic deaths in the world.^1^ In 2017, Malawi’s gross domestic product was 8.3 billion United States dollars (US$) and its gross national income per capita was US$ 620.^6^ Donors contributed to 55% of the Malawian health ministry’s budget during 2022–2023. This reliance on donations raises concerns over the capacity to increase physical access to health facilities across the country.^7^

With only 14 orthopaedic surgeons for a population of 20 million (in 2021), most fracture care in Malawi is provided by orthopaedic clinical officers (non-physicians who undergo 3 years’ training towards an orthopaedic diploma).^8^ In district (secondary) hospitals, such orthopaedic clinical officers predominantly provide non-operative care; tertiary hospitals on the other hand are staffed by orthopaedic surgeons and are therefore able to provide operative care.^8^

Most trauma and fracture interventions in low- and middle-income countries only report improvement in knowledge and behaviour – to date there has been very little evidence for whether this translates into improvement in clinical processes and patient outcomes.^9^ For example, despite implementation interventions using the World Health Organization (WHO) Surgical Safety Checklist showing sustainable national scale-up in Benin, Cameroon, and Madagascar, no clinical outcomes were reported.^10^^,^^11^ However, it should be noted that implementation of interventions such as the checklist have led to a reduction in mortality in low- and middle-income countries.^12^

Our primary aim was to evaluate patient function 1 year after an open tibia fracture before and after the implementation of a quality improvement intervention. Our secondary aim was to identify barriers and facilitators to the success of the open fracture quality improvement intervention.

## Methods

The study adhered to standards for reporting implementation studies.^13^

### Study protocol

This implementation study was nested in a prospective multicentre cohort study conducted in six hospitals in Malawi and was conducted according to the previously published protocol.^14^

### Ethics approval

The study was approved by the College of Medicine Research and Ethics Committee (COMREC Ref number: P.09/20/3130) in Malawi, and the Liverpool School of Tropical Medicine (Reference number: 20–068) in England. All patients in the study provided written informed consent and all participating health-care providers provided verbal informed consent.

### Study site

The hospitals included two tertiary hospitals (Queen Elizabeth Central Hospital and Kamuzu Central Hospital) and four district hospitals (Dedza District Hospital, Ntcheu District Hospital, Balaka District Hospital and Machinga District Hospital).

### Study population

Between February 2021 and March 2022, we recruited patients who were 18 years or older and had open tibia fractures classified as *Arbeitsgemeinschaft für Osteosynthesefrage* Foundation/Orthopaedic Trauma Association class 42.^15^ The Gustilo classification of each fracture^16^ was recorded by the most senior surgeon or orthopaedic clinical officer at each site during debridement. Sex was reported by patients and recorded by trained research assistants.

Clinical knowledge and implementation evaluation was captured on health-care providers that attended the Malawi Orthopaedic Association’s annual general meetings on 23–24 September 2021 and 23 September 2022.

### Intervention bundle

#### Components of the bundle

The quality improvement intervention for open fractures (hereafter referred to as the intervention or the intervention bundle) consisted of core and adaptable components (Box 1). All intervention bundle costs (i.e. development, materials, venue fees, transportation and allowances) were funded by international donors and, in 2019 the cost of the whole intervention totalled to US$ 26 500.

Box 1 **Core and adaptable components of the open fracture intervention bundle**

***Core components:***

- A hospital management protocol for all open fractures was implemented at first and second level health-care facilities based on the 17 Malawi Orthopaedic Association/AO (*Arbeitsgemeinschaft für Osteosynthesefragen*) Alliance guidelines.^5^ This protocol included administering early antibiotics, photographing the wound and performing debridement in theatre with adequate anaesthesia.
- A national educational course for orthopaedic clinical officers on the open fracture guidelines.
- Improved documentation of each guideline by using standard proformas for open fracture management.
- A letter signed by hospital director and the chair of the Malawi Orthopaedic Association encouraging that open fractures should be debrided in theatre with spinal or general anaesthesia.
- Donation of one external fixator set to each district hospital.

***Adaptable components:***

- Dissemination of guidelines via posters in all the primary and secondary centres that may refer to tertiary centres.
- In addition to the above, sites were visited to select referral centres for education and training to enhance the implementation of the protocol. Visits and training were undertaken by local multidisciplinary members and the principal investigator.
- Health-care telephone contacts were shared to improve communication of referrals to tertiary centres.
- Feedback for the health-care professionals was offered in the first 3 months after the course on pre- and post-debridement photos.

#### Intervention bundle development

As part of the implementation strategies^17^ to bring all relevant stakeholders together, two consensus groups (consisting of expert Malawian orthopaedic clinical officers, orthopaedic surgeons and international orthopaedic teaching faculty) were convened 2 years before the implementation to agree on 17 guidelines for open fracture management. Additionally, one year before the implementation, the stakeholders agreed on the material and methods to be included in a training course on open fracture management.^5^^,^^18^ Although the expert groups helped create the quality improvement intervention bundle, no implementation material was distributed to clinical officers or the hospital management team before the implementation period.

### Implementation

The intervention bundle was introduced into clinical practice midway through the prospective cohort study, during 23–24 September 2021.

### Assessment of patient function

Patients reported their musculoskeletal function at baseline (asking participants to report their pre-injury score) and 6 weeks, 3 months, 6 months and 1 year after injury, using the short musculoskeletal functional assessment questionnaire. This assessment provides a dysfunction score, which ranges from 0 (no functional impairment) to 100 (severe functional impairment).^19^^,^^20^

### Assessment of clinical knowledge

Health-care providers taking the open fracture course completed an online questionnaire at three timepoints: at the start of the course; immediately after taking the course; and 1 year after taking the course. The questionnaire included questions relating to the participants’ demographic characteristics plus 20 knowledge-based questions on the open fracture standards and guidelines. This questionnaire also contained questions regarding implementation evaluation outcomes, which followed Proctor’s framework and included the acceptability score, appropriateness score, feasibility score and normalization (understanding/opinion) questionnaire.^21^^,^^22^

### Assessment of clinical management

Trained research assistants documented treatment characteristics based on information in medical notes during the initial hospital admission using electronic tablets and secure servers.

### Statistical analysis

Our target sample size for the primary outcome (patient function) was 160 participants (80 before and 80 after implementation of the intervention). This sample size would provide 80% power to detect a 20% variance in the short musculoskeletal function assessment index score before the intervention bundle was implemented compared with after, with *α* of 0.01; allowing for 20% loss to follow-up. We planned to continue recruitment until the end of the study period (1 year) even if we exceeded our target.

We compared baseline demographics and clinical management for patients with open tibia fractures before and after implementation of the intervention bundle using two comparative statistical tests: Kruskal–Wallis and χ2.

To investigate trajectories over time in patient function, in clinical knowledge, and in implementation evaluation following implementation of the open fracture interventional bundle, we constructed Bayesian multilevel regression models, with inference drawn using Markov chain Monte Carlo sampling.

For functional outcomes we fitted a model for hospital setting and Gustilo grade to account for the fact that the open fracture guidelines are guided by setting (district or tertiary hospital) and injury severity (Gustilo grade). Models were fitted using the R brms package as an interface to CmdStanR in R (R Foundation, Vienna, Austria).^23^ We modelled short musculoskeletal function assessment scores and knowledge scores using a Gaussian distribution and an ordinal model for the implementation evaluation outcomes, which included participant-level random intercepts. We summarized posterior distributions to compare the effects before and after implementation of the interventional bundle, stratified by hospital setting and Gustilo classification on short musculoskeletal function assessment over 1 year. Similarly, using posterior distributions, we compared knowledge and implementation evaluation outcomes before, immediately after and 1 year after implementation of the intervention bundle. Code and data are available in our online respository.^24^

## Results

### Study population demographics

Baseline demographic characteristics of patients are outlined in Table 1. We recruited a total of 287 adult patients with open tibia fractures between February 2021 and March 2022: 162 patients were recruited before implementation of the open fracture intervention bundle and 125 patients after. Thirty-six (22%) pre-intervention patients and 27 (22%) post-intervention patients received treatment in district hospitals. No differences were found in terms of age (*P*-value: 0.70), sex (*P*-value: 0.86) or Gustilo grading (*P*-value: 0.46) between the before and after groups.

**Table 1:** Baseline demographics of patients with open tibia fractures, Malawi, 2021–2022

Characteristic	No. (%)^a^		*P*
	Pre-intervention (*n* = 162)	Post-intervention (*n* = 125)	
**Age in years (IQR)**	33.5 (26.0–44.8)	34.0 (26.0–44.0)	0.70
**Male**	139 (86)	109 (87)	0.86
**Treated in district hospitals **	36 (22)	27 (22)	1.00
**Injury severity^b^**			0.46
Gustilo type I/II	96 (60)	67 (55)	
Gustilo type III	64 (40)	55 (45)	

IQR: interquartile range.

^a^ Values are numbers and percentage; if not, another unit is indicated.

^b^ Five (2%) fractures missing Gustilo classification.

### Patient function

There was no difference in empirical median short musculoskeletal function assessment dysfunction scores at 1 year, 6.8 (interquartile range, IQR: 1.5 to 14.5) before intervention, compared to 8.4 (IQR: 3.8 to 23.2) after intervention.

**Fig. 1:**
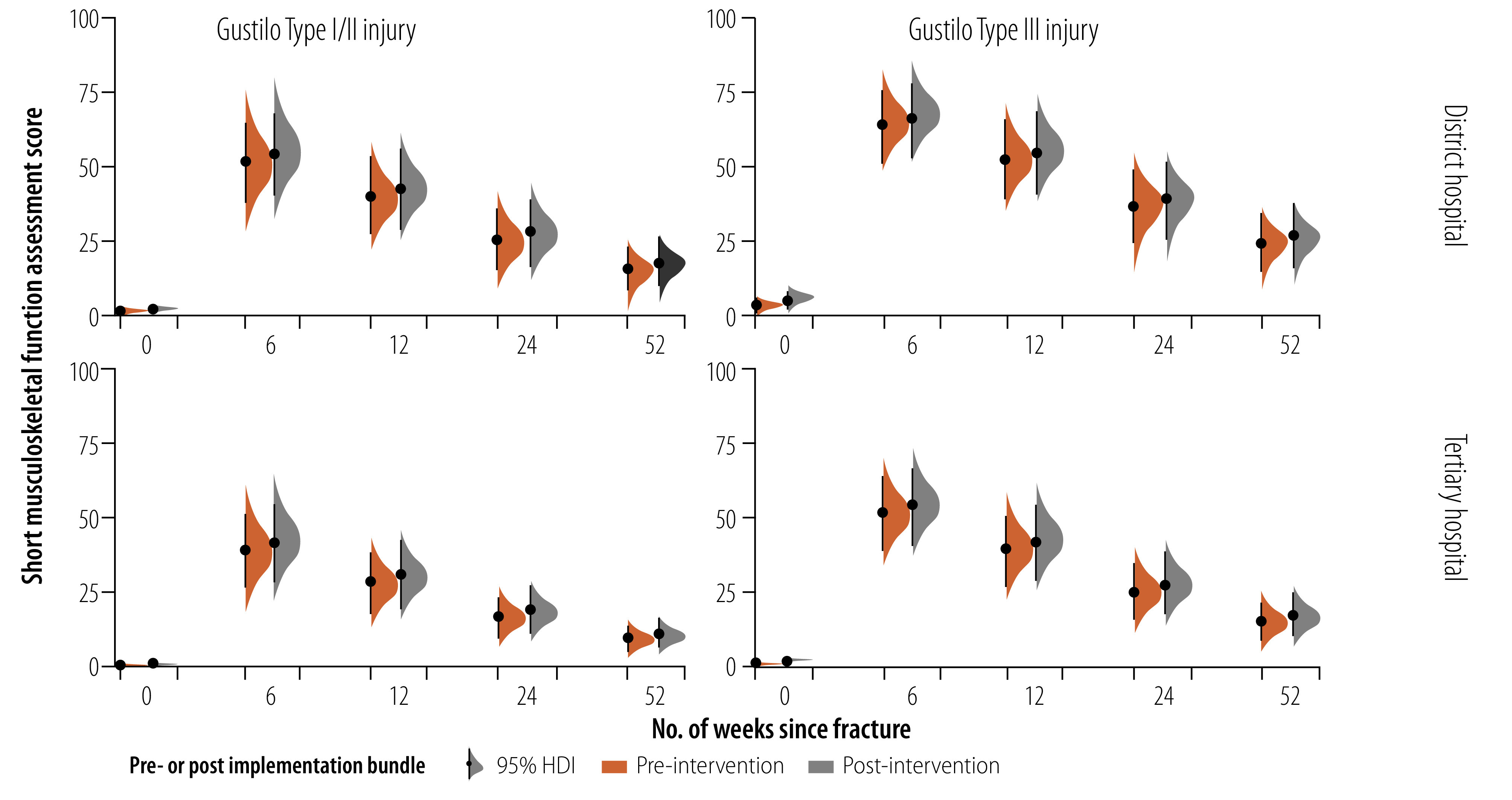
Functional score of patients with open tibia fracture, pre- and post-intervention, Malawi, 2021–2022

There was weak evidence of worsening modelled short musculoskeletal function assessment score at 1 year for participants in district hospitals with Gustilo I/II injuries (mean posterior difference short musculoskeletal function assessment: 1.9; 95% confidence interval, CI: −0.2 to 4.4) and with Gustilo III injuries (mean posterior difference short musculoskeletal function assessment: 2.4; 95% CI: −0.3 to 5.5) (Fig. 1).

HDI: highest density interval.

Similarly, there was weak evidence of worsening modelled short musculoskeletal function assessment score at 1 year for participants in tertiary hospitals with Gustilo I/II injuries (mean posterior difference short musculoskeletal function assessment: 1.7; 95% CI: 0.2 to 3.6) and with Gustilo III injuries (mean posterior difference short musculoskeletal function assessment: 2.4; 95% CI: 0.2 to 4.7).

### Clinical knowledge 

A total of 57 candidate health-care providers attended the open fracture course; their demographics are summarized in the online repository.^24^

**Fig. 2:**
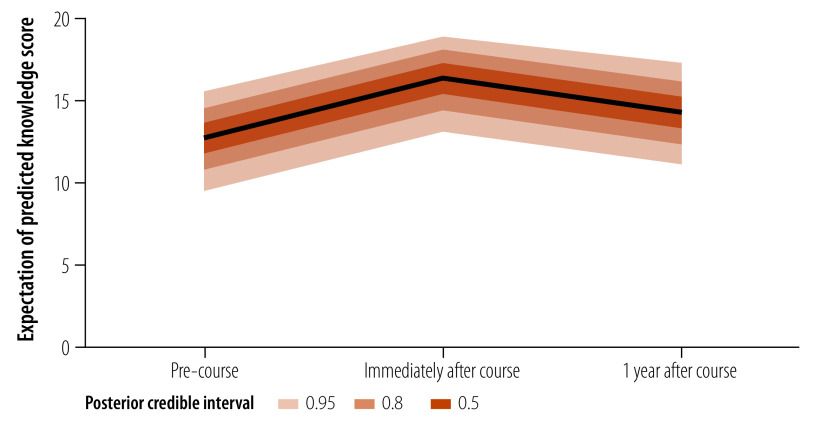
Trends in knowledge scores of health-care providers attending an open fracture course, Malawi, 2021–2022

Note: A total of 57 health-care providers attended the course. The data include participant-level random effects.

Our results suggest that open fracture knowledge scores (out of 20) might have improved both immediately after the course (posterior mean: 17.1, 95% credible interval: 14.6 to 18.25) and 1 year after the course (posterior mean: 14.8, 95% credible interval: 13.9 to 15.7) compared to before the course (posterior mean: 13.4, 95% credible interval: 11.2 to 14.6), however, the credible intervals overlap (Fig. 2).

### Clinical management

Results of documentation and clinical management (clinical processes) by the health-care providers are summarized in Table 2.

**Table 2:** Clinical process pre- and post-intervention for open tibia fractures, Malawi, 2021–2022

Clinical process	All hospitals (*n* = 287)				Tertiary hospitals (*n* = 224)				District hospitals (*n* = 63)		
	No./eligible patients (%)		*P*		No./eligible patients (%)		*P*		No./eligible patients (%)		*P*
	Pre	Post			Pre	Post			Pre	Post	
Documented time to antibiotics^a^	65/160(40)	49/125 (39)	0.48		43/122 (35)	35/98 (36)	0.44		20/36 (56)	14/27(52)	0.37
Documented neurovascular status^a^	58/160 (36)	29/125 (23)	0.01		45/126 (36)	12/98 (12)	< 0.01		13/36 (36)	17/27 (63)	0.07
Splinting of limb before transfer to the theatre or ward	150/162 (93)	116/125 (93)	0.18		117/126 (93)	90/98 (92)	0.16		33/36 (92)	25/27 (93)	0.10
Adequate timing to debridement^b,c^	33/57 (58)	12/34 (35)	0.05		20/39 (51)	4/21 (19)	0.03		13/18 (72)^a^	8/13 (62)	0.70
Debridement under general or spinal anaesthetic	117/160 (73)	99/125 (79)	0.07		113/126 (90)^d^	88/98 (90)^d^	0.75		4/34 (12)	11/27 (41)	0.02
External fixation for Gustilo III injuries	21/64 (33)	29/53 (55)	0.02		21/57 (37)	28/50^e^ (56)	0.28		0/10 (0)	1/4 (25)	0.06
Referral of Gustilo III injuries to tertiary hospitals	NA	NA	NA		NA	NA	NA		5/10 (50)	3/4 (75)	0.10
Definitive fixation within 72 hours	49/110 (45)	53/95 (56)	0.14		49/110 (45)	53/95 (56)	0.14		NA	NA	NA

NA: not applicable.

^a^ Two patients were missing hospital setting or timing of intervention.

^b^ Within 12 hours for Gustilo II or III and within 24 hours for Gustilo I injuries (based on the open fracture guidelines).^5^

^c^ Forty percent missing timing of debridement.

^d^ Debridement with inadequate anaesthetic occurred in district hospitals before referral to a tertiary hospital.

^e^ One participant from a district hospital was referred to a tertiary hospital for external fixation.

We observed no differences between pre- (baseline) and post-intervention in documentation of antibiotics (65/160; 40% versus 49/125; 39%; *P*-value: 0.48); splinting of the limb before transfer to the theatre or ward (150/160; 93% versus 116/125; 93%; *P*-value: 0.18) or definitive fixation within 72 hours (49/110; 45% versus 53/95; 56%; *P*-value: 0.14).

Poorer levels of documentation of neurovascular status were observed post-intervention (58/160; 37% pre- versus 29/125; 23% post-intervention; *P*-value: 0.01). Adequate timing to debridement (within 12 hours for Gustilo II/III injuries and within 24 hours for Gustilo I injuries) also worsened (33/57; 58% pre- versus 12/34; 35% post-intervention; *P*-value: 0.05).

There was no significant change in instances of external fixation in district hospitals (0/10; 0% pre- versus 1/4; 25% post-intervention; *P*-value: 0.06) and in referral of Gustilo III injuries from district hospitals (5/10; 50% pre- versus 3/4; 75% post-intervention; *P*-value: 0.10), although we note these two comparisons were based on small numbers. The number of open tibia fracture debridement under general or spinal anaesthetic in district hospitals improved (4/34; 12% pre- versus 11/27; 41% post-intervention; *P*-value: 0.05).

### Evaluation of training

**Fig. 3:**
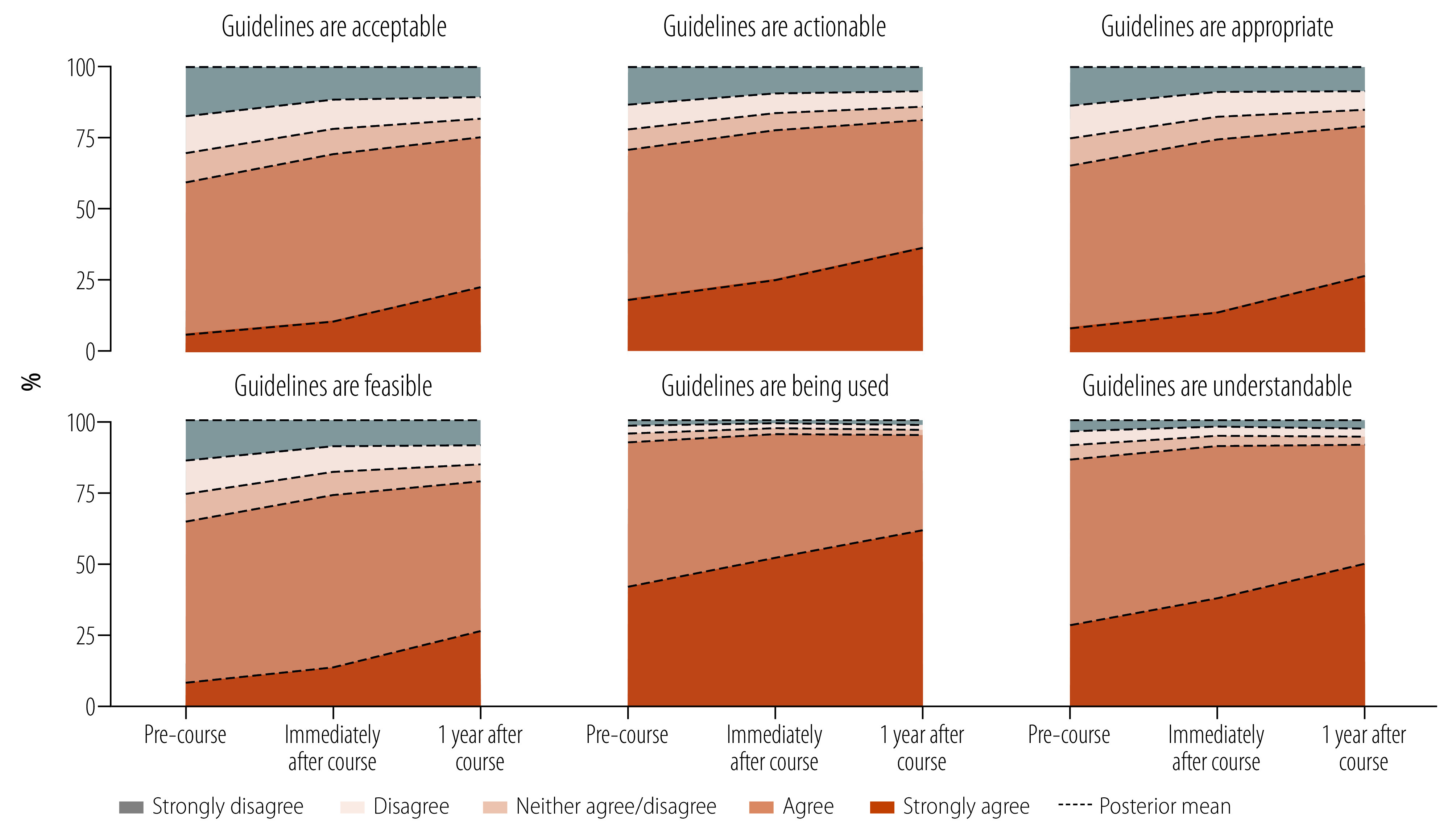
Implementation evaluation outcome responses by health-care providers attending an open fracture course, Malawi, 2021–2022

Note: A total of 57 health-care providers attended the course. Estimated from a Bayesian multilevel item response model with nest effects for domains and items, and participant-level random effects.

Overall, all implementation evaluation outcomes improved 1 year post-intervention (Fig. 3), with the lowest improvement following implementation being acceptability (agreed mean posterior distribution: 52.2%; 95% highest density interval: 0.10% to 65.0%; strongly agreed: 23.3%; 95% highest density interval: 0.03% to 79.0%) and the highest being participation (agreed: 33.7%; 95% highest density interval: 0.1% to 64.2%; strongly agreed: 61.2%; 95% highest density interval: 0.62% to 98.8%).

## Discussion

Our study shows that while implementation of the open fracture interventional bundle in district hospitals might have led to a sustained improvement in clinical knowledge and positive evaluation of the intervention, there was little overall change in clinical management and weak evidence of worsening patient function. For example, we show that only 11 of 27 patients in district hospitals with open fractures had debridement under spinal or general anaesthesia after implementation of the intervention bundle.

One reason for this lack of change in management and patient outcome may be inadequate hospital facilities and resources, a recognized challenge for implementation studies.^25^^,^^26^ Challenges relating directly to implementing open fracture guidelines in settings such as government hospitals in Malawi include a severe lack of resources such as water, electricity, radiography, basic orthopaedic equipment, anaesthesia and operating theatres.^27^^,^^28^ In addition, other specialities, such as obstetrics, might be prioritized over trauma operations.^29^

Possible explanations for the worsening of patient-reported function following treatment in tertiary hospitals in the post-intervention period could be increased infections during Malawi’s wet season (November–March), or increased road traffic injuries during the Christmas holiday period due to more patients with injuries and delays to treatment.^30^^,^^31^ Solutions could include improving infection control in hospitals, increasing road safety awareness campaigns and strengthening law enforcement.^31^

While better overall surgical care and better facilities and resources in district hospitals are needed to improve patient outcomes following trauma, Malawi faces challenges. The country is heavily dependent on donors, and to date development assistance for low-income countries has been heavily skewed towards communicable diseases; only 0.6% of funding has been allocated to injury and violence.^32^ Those challenges are combined with a rapidly increasing burden of injuries. While improving surgical care is undoubtedly costly (an estimated US$ 23 billion annual investment between 2015–2030 would be needed to reach safe levels of global surgical care),^33^ the economic loss for people with surgical conditions is estimated to be much more, around US$ 12.3 trillion among low- and middle-income countries between 2015–2030.^34^ Therefore, substantial investment from government and donors in surgical care and road safety prevention will be required to reduce the disability from road-traffic injuries victims.

Other barriers to successful implementation could include health-care provider behaviour, task-shifting (when orthopaedic clinical officers are required to provide fracture care due to the low ratio of doctors per population in Malawi), poor supervision and lack of feedback from specialists.^26^^,^^33^^,^^35^^,^^36^ We found that after the intervention, only 39% (49/125) of health-care providers documented the timing of antibiotics, and only 23% (29/125) documented neurovascular status in the medical notes. Qualitative evidence from clinical officers in district hospitals in low-income countries, and informal discussions with clinical officers during our study suggest that poor motivation is also a barrier to the operative care of patients, which could partly be due to inadequate continuous education, poor career progression, and lack of supervision and accountability.^37^

A systematic review of approaches to motivate physicians and nurses in low- and middle-income countries found that studies that primarily focused on financial compensation either showed no or negative effects; interventions with a supervisory component, on the other hand, had mostly positive effects.^38^ Different supervision models for surgical care in low- and middle-income countries have been proposed, including managed surgical networks;^39^ national accountability and monitoring programmes;^40^ and regular in-person site visits by surgical specialists.^41^ Both the training of orthopaedic specialists and orthopaedic clinical officers are cost-effective and are required to improve supervision.^42^^,^^43^Nevertheless, district hospitals should ideally establish referral pathways to tertiary hospitals where patients can access adequate expertise and resources to treat their complex injuries.

Despite the burden of injury rapidly worsening and task-shifting being common in many low- and middle-income countries, it is unclear if our identified barriers can be generalized to other low- and middle-income countries where other factors may come into play. For example, health-care systems in some low-and middle-income countries have higher physician-per-population ratios, with increased health-care resources,^44^ than in Malawi; this difference could reduce the suggested barriers of poor hospital environment and lack of specialist supervision. Additionally in some countries, particularly in the Saharan belt, training of traditional bone setters might help reduce complications of open fracture care.^45^

The study has some limitations. First, 78% (224/287) of participants were from tertiary hospitals (which have permanent orthopaedic specialists); as such, the change in clinical process specific to district hospitals (which rely on orthopaedic clinical officers) might have been overshadowed. More participants from district hospitals may help detect a change in patient function after intervention. Second, health-care providers participating in our study were only recruited up to 6 months after the intervention period; it is possible that changes in implementation evaluation outcomes might take longer to translate into changes in clinical process and patient outcomes. While uncertainty intervals for knowledge and behaviour changes overlapped, our findings did demonstrate a strong trend towards improved knowledge after the course. Third, the before-and-after study design might have introduced confounders, selection bias, or been influenced by the Hawthorne effect (where participants change their behaviour in response to being observed).^46^ Finally, this study was conducted during the coronavirus disease 2019 (COVID-19) pandemic, which may have reduced monitoring in district hospitals and reduced theatre capacity (for example, due to anaesthesia redeployment to high-dependency units).^47^

Further studies aimed at investigating if an open fracture interventional bundle is effective in changing clinical processes and patient outcomes following open tibia fractures in Malawi (or in other low- and middle-income countries) should aim to recruit a greater number of participants from district hospitals over a longer period. Additional studies could focus on improving orthopaedic facilities and resources in hospital; improving orthopaedic clinical officer motivation; and encouraging teamwork between orthopaedic clinical officers and orthopaedic surgeons. 

Future implementation studies could evaluate whether managed surgical networks, orthopaedic clinical officer accountability, and district hospital visits from orthopaedic surgeons might improve outcomes following open fractures in low- and middle-income countries. Training district health system managers in clinical governance may increase their monitoring evaluation skills in low- and middle-income countries. Meanwhile, financial loans to health-care workers to undertake training courses might be an effective way of improving training while also mitigating the loss of skilled orthopaedic personnel to other countries.^48^

In summary, our study shows that in Malawi, implementation of an open fracture interventional bundle did not improve functional outcomes for patients with an open tibia fracture, despite possible improvement in clinician knowledge. This result could be due to a severe lack of resources in hospital and/or poor supervision of health-care providers. Increases in injuries during the rainy season and public holidays may also have affected the study outcome. 
